# CircETFA upregulates CCL5 by sponging miR-612 and recruiting EIF4A3 to promote hepatocellular carcinoma

**DOI:** 10.1038/s41420-021-00710-x

**Published:** 2021-10-29

**Authors:** Chen Lu, Dawei Rong, Bingqing Hui, Xuezhi He, Wei Jiang, Yetao Xu, Hongyong Cao, Zekuan Xu, Weiwei Tang

**Affiliations:** 1 Department of General Surgery, The First Affiliated Hospital of Nanjing Medical University, Nanjing, Jiangsu China; 2 Department of clinical medicine, The First Clinical Medical College of Nanjing Medical University, Nanjing, Jiangsu China; 3 School of Medicine, Southeast University, Nanjing, Jiangsu China; 4 Hepatobiliary/Liver Transplantation Center, The First Affiliated Hospital of Nanjing Medical University, Nanjing, Jiangsu China; 5 Department of Oncology and cancer rehabilitation center, The First Affiliated Hospital of Nanjing Medical University, Nanjing, Jiangsu China; 6 State Key Laboratory of Reproductive Medicine, The Research Center for Bone and Stem Cells, Department of Anatomy, Histology and Embryology, Nanjing Medical University, Nanjing, Jiangsu China; 7 Department of General Surgery, Nanjing First Hospital, Nanjing Medical University, Nanjing, Jiangsu China; 8 Department of Obstetrics and Gynecology, The First Affiliated Hospital of Nanjing Medical University, Nanjing, Jiangsu China

**Keywords:** miRNAs, Oncogenes, Cancer epidemiology

## Abstract

As a kind of malignant tumors, hepatocellular carcinoma (HCC) has been studied continuously, but the mechanisms are not well understood. Circular RNAs (circRNAs) are widespread in eukaryotes and play an important role in the growth of organisms and in the occurrence of diseases. The role of circRNAs in HCC remains to be further explored. In this study, CircRNA microarray analysis was used to assess the plasma from HCC patients and healthy controls and to identify circRNAs involved in HCC tumorigenesis. CircETFA was overexpressed in HCC tissues, plasma, and cells. Clinicopathological data revealed that abnormally high circETFA expression was associated with a poor prognosis. In function, circETFA promotes the malignant phenotype of HCC cells in vivo and in vitro, inhibits cycle arrest, and decreases the proportion of apoptotic cells. In mechanism, it can upregulate C-C motif chemokine ligand 5 (CCL5) in HCC cells, thereby regulating the phosphoinositide 3-kinase (PI3K)/Akt pathway and other key downstream effectors (e.g., FoxO6). Furthermore, circETFA prolonged the half-life of CCL5 mRNA by recruiting the eukaryotic initiation factor 4A3 (EIF4A3) and acted as a sponge of hsa-miR-612 to suppress the silencing effect of hsa-miR-612 on CCL5. In conclusion, CircETFA can increase the expression of CCL5 to promote the progression of HCC by sponging hsa-mir-612 and recruiting EIF4A3, and is promising as a novel biomarker and therapeutic target.

## Introduction

Primary liver cancer is a worldwide problem, ranking fourth among the cancer-related deaths [1]. HCC is the most common type of primary liver cancer, and hundreds of thousands of people die from HCC every year [2, 3]. Despite multiple treatment options, the 5-year survival rate for HCC remains very low [4]. In addition, recurrence and metastasis are major challenges in the treatment of liver cancer [5]. In the process of metastasis, frequent intrahepatic and extrahepatic metastases lead to poor prognosis [6]. This is because the molecular pathogenesis of HCC remains unclear [7]. Therefore, understanding the basic biological principles of HCC and developing innovative therapies has important clinical significance [8].

In the past, non-coding RNAs have always been the topic of research in the RNA field [9]. With the development of sequencing technology, circRNAs have received a lot of attention as a new type of non-coding RNA [10]. CircRNAs are more stable and do not easily degrade owing to their special closed-ring structure [11, 12]. CircRNAs are involved in a variety of physiological and pathological processes [13], and the abnormal regulation of circRNAs is related to many diseases [14–16]. For example, circNDUFB2 inhibits the progression of non-small cell lung cancer, and circSDHC promotes the progression and metastasis of renal cell carcinoma [17, 18]. Studies on the mechanism of circRNAs mainly focus on sponging microRNA (miRNA), interacting with RNA binding proteins (RBPs), and translating into peptides [19–21]. For example, circCUL2 regulates the malignant transformation and cisplatin resistance of gastric cancer by sponging mir-142-3p [22]. CircPCNX binds to and regulates the function of the RBP AUF1 and regulates cell proliferation [23]. Additionally, circSMO encodes 193aa and regulates the progression of glioblastoma [24]. In addition, circRNAs can be detected in body fluids; therefore, they are expected to become promising biomarkers [25]. However, it is not clear what role circRNA plays in HCC.

In this study, we performed a circRNA microarray analysis of the plasma from patients with HCC. We aimed to identify differentially expressed circRNAs in HCC and explore their functions and potential mechanisms to provide novel insights into the occurrence and development of HCC.

## Results

### CircETFA is identified as a circular RNA associated with HCC

**Fig. 1:**
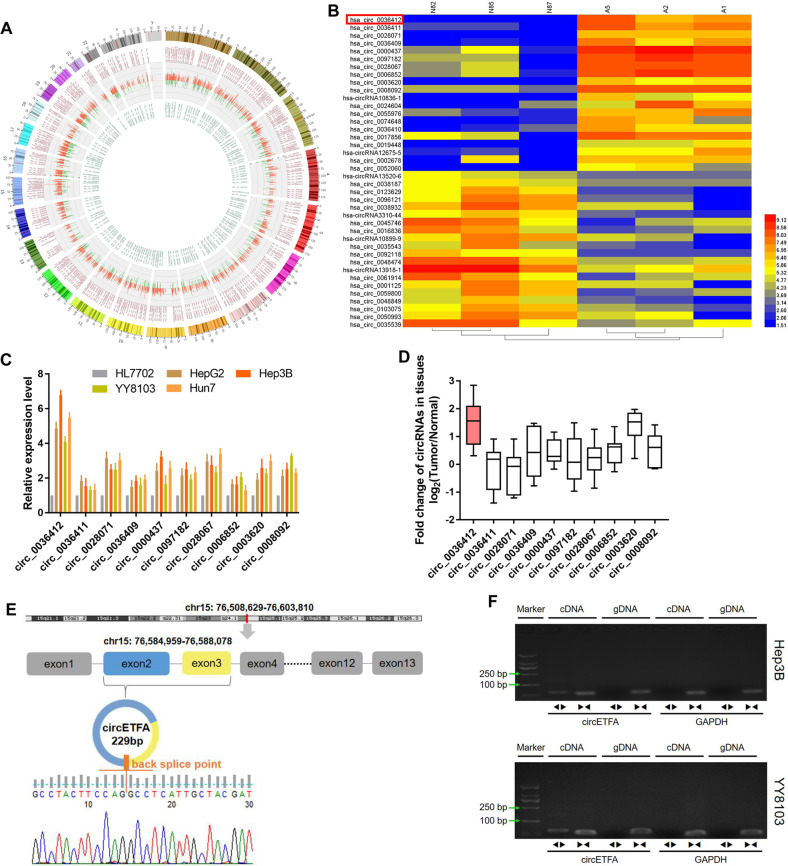
CircETFA is overexpressed in HCC tissues and cells. **A** circRNA microarray analysis with three pairs of total RNA from the plasma of HCC patients and healthy controls identified a total of 5122 differentially expressed circRNAs. **B** The heat map shows the normalized and log_2_-transformed signal values of the 40 circRNAs with the most obvious differential expression. **C**, **D** qRT-PCR detected expression of the most significant up-regulated ten circRNAs in HCC cell lines and tissues. **E** Sanger sequencing showed the splicing region of circETFA head and tail and determined its position and sequence in the genome. **F** RT-PCR excluded genome rearrangement. The data are shown as mean ± SD. *P < *0.05*, **P < 0.01, ***P < 0.001.

To identify circRNAs involved in HCC tumorigenesis, we performed circular RNA microarray analysis on three pairs of total RNA from the plasma of HCC patients and healthy controls. A total of 5122 differentially expressed circRNAs were identified (fold change (FC) ≥ 2 or FC ≤ −2, *P*-value ≤ 0.05) (Fig. 1A). Among them, 4162 circRNAs were significantly upregulated, and 960 circRNAs were significantly downregulated (Fig. S1A). The complete expression profiles are presented in Supplementary Table S1 and have been uploaded to the GEO platform (ID: GSE166678). In addition, we generated a heat map of the 40 circRNAs with the most obvious expression differences in the microarray results to show the normalized and log_2_-converted signal values of these circRNAs (Fig. 1B). We paid special attention to the abnormally highly expressed circRNAs because they are more helpful for disease diagnosis and treatment. As shown in Supplementary Table S1, the top ten circRNAs with the most upregulation were only derived from two parental genes (ETFA and CORO1C), which are involved in increased susceptibility to certain tumors [26–29]. Subsequently, we detected the expression of these circRNAs in human HCC cell lines and tissues. The results of qRT-PCR showed that hsa_circ_0036412 had the highest expression levels in HCC cells and tissues (Fig. 1C, D). Hsa_circ_0036412 (referred to as circETFA) is 229 bp in length and is produced by the ETFA gene. It was spliced from exons 2 and 3 from the beginning to the end (Fig. 1E). To substantiate the circular form of circETFA, we first performed Sanger sequencing on the qRT-PCR product to detect the splicing region of circETFA (Fig. 1E). Next, to exclude head-to-tail splicing from trans-splicing or genome rearrangement, we designed two sets of primers, divergent and convergent primers, to perform RT-PCR (Fig. 1F). In addition, we found that circETFA had obvious resistance to RNase R through the RNase R tolerance test (Fig. S1B). These results proved that circETFA was a true circRNA and was highly expressed in HCC.

### Expression and characterization of circETFA are determined in HCC

**Fig. 2:**
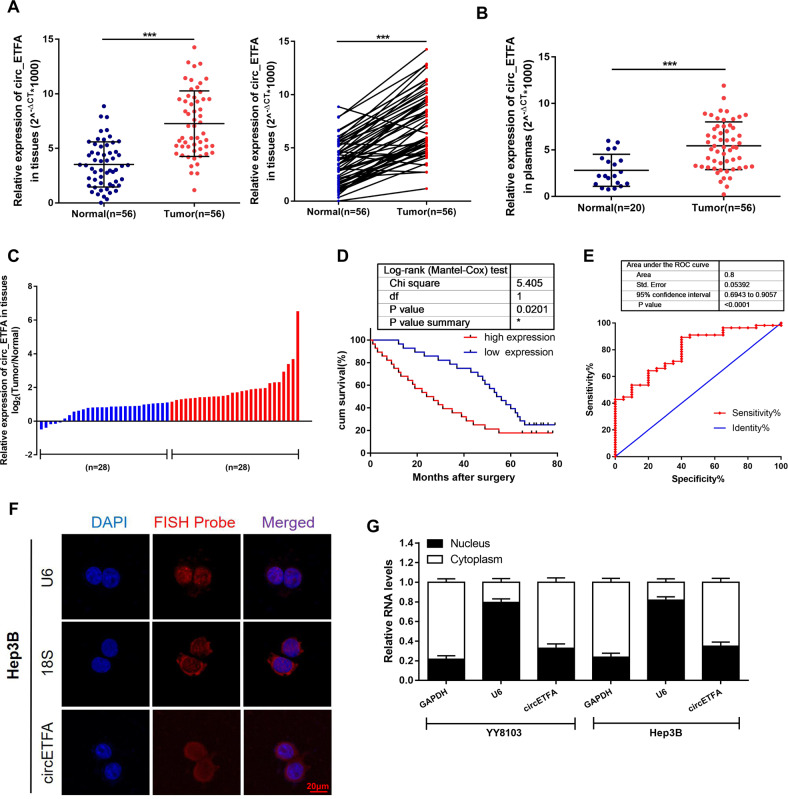
Expression and characterization of circETFA in HCC. **A**, **B** CircETFA expression was detected in the tumor tissues and plasma of HCC patients. **C** The patients were stratified into high and low circETFA groups using median circETFA value as a cutoff value. **D** Relationship between the expression of circETFA and the OS rate of HCC patients. **E** The area under the ROC curve of circETFA. **F**, **G** FISH and nucleoplasm separation experiment was performed to detect subcellular localization of circETFA in Hep3B. The data are shown as mean ± SD. *P < *0.05*, **P < 0.01, ***P < 0.001.

To further examine the correlation between circETFA and HCC, we tested the expression of circETFA in the tumor tissue and plasma of 56 HCC patients. The results showed that circETFA was overexpressed in the tumor tissue and plasma of patients with HCC (Fig. 2A, B). According to the median, patients were divided into the high circETFA group (*n* = 28, circETFA expression> median) and low circETFA group (*n* = 28, circETFA expression <median) (Fig. 2C). Kaplan-Meier analysis showed that circETFA negatively correlated with the overall survival (OS) rate (Fig. 2D). Additionally, the area under the receiver operating characteristic (ROC) curve of circETFA, which distinguishes HCC plasma from normal plasma, was 0.8 (Fig. 2E). These results indicate that circETFA is highly expressed in HCC and negatively correlated with prognosis.

### CircETFA promotes proliferation, invasion, and metastasis in HCC cells in vitro

**Fig. 3:**
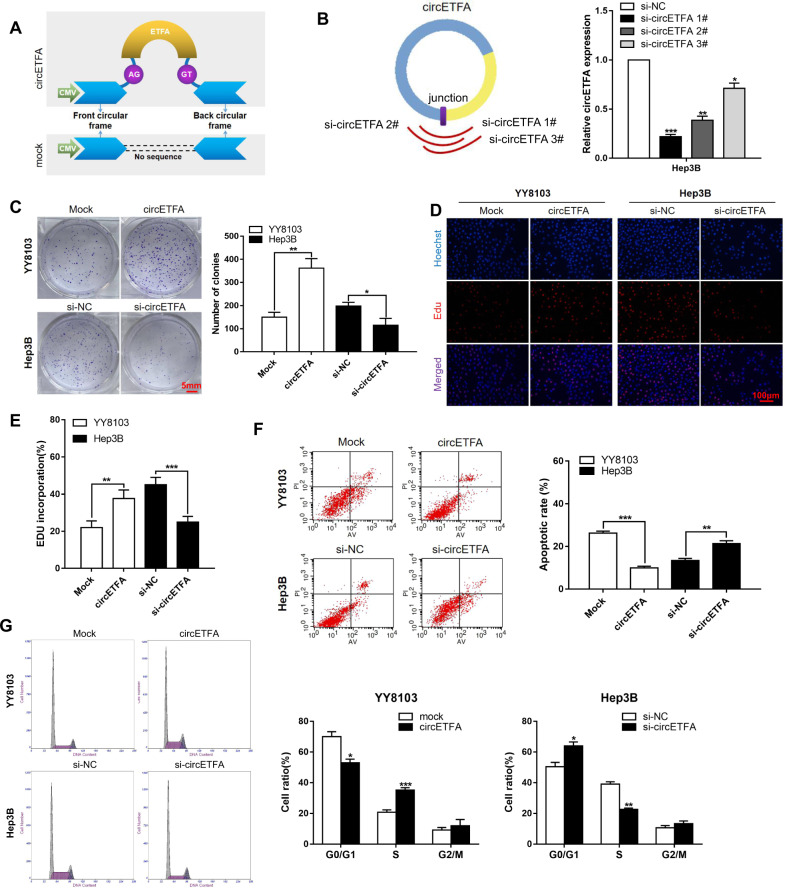
CircETFA alters proliferation, apoptosis, and the cell cycle in HCC cells. **A** A schematic diagram of the overexpression vector construction of circETFA. **B** A schematic diagram of design of circETFA small interfering RNA and test result of knockdown efficiency. **C**–**E** Clone formation experiments and EdU assays were used to detect the influence of circETFA on HCC cell proliferation. **F** The effect of circETFA on apoptosis was analyzed by flow cytometry. **G** The effect of circETFA on the cell cycle was analyzed by flow cytometry. The data are shown as mean ± SD. *P < *0.05*, **P < 0.01, ***P < 0.001.

In order to determine the distribution of circETFA, we performed fluorescence in situ hybridization (FISH) and nuclear-cytoplasmic separation experiments and found that circETFA was distributed in the nucleus and cytoplasm, mainly in the cytoplasm (Fig. 2F, G). To study the biological function of circETFA in HCC, the ectopic expression of circETFA was achieved using a lentiviral infection system (Fig. 3A) to establish YY8103 cell line stably expressing circETFA (Fig. S1C). At the same time, we used siRNAs to knockdown circETFA. To ensure effectiveness, we designed three siRNAs targeting different sites to silence the expression of circETFA in Hep3B cells. Si-circETFA 1# had the highest silencing efficiency and was used in subsequent experiments (Fig. 3B). The selection of overexpression and knockdown cell lines was based on the expression level of circETFA in the cell lines (Fig. 1C). In addition, we observed that overexpression or silencing of circETFA did not change the linear ETFA levels in HCC cells (Fig. S1D).

The CCK-8 analysis showed that overexpression of circETFA increased HCC proliferation, and knockdown of circETFA inhibited HCC proliferation (Fig. S1E–F). The same results were obtained in the clone formation assay and EdU experiments (Fig. 3C–E). In addition, we used flow cytometry to explore the effects of circETFA on the cell cycle and apoptosis. The results showed that overexpression of circETFA inhibited cell apoptosis, increased the proportion of S-phase cells, and decreased the ratio of G0/G1 (Fig. 3F, G). At the protein level, overexpression of circETFA significantly suppressed apoptosis-related proteins, such as cleaved caspase-3 and elevated the levels of G1/S-phase checkpoint proteins, including CyclinE1 and CyclinD1 (Fig. S1G). However, circETFA knockdown resulted in the opposite result (Figs. 3F, G and S1G).

**Fig. 4:**
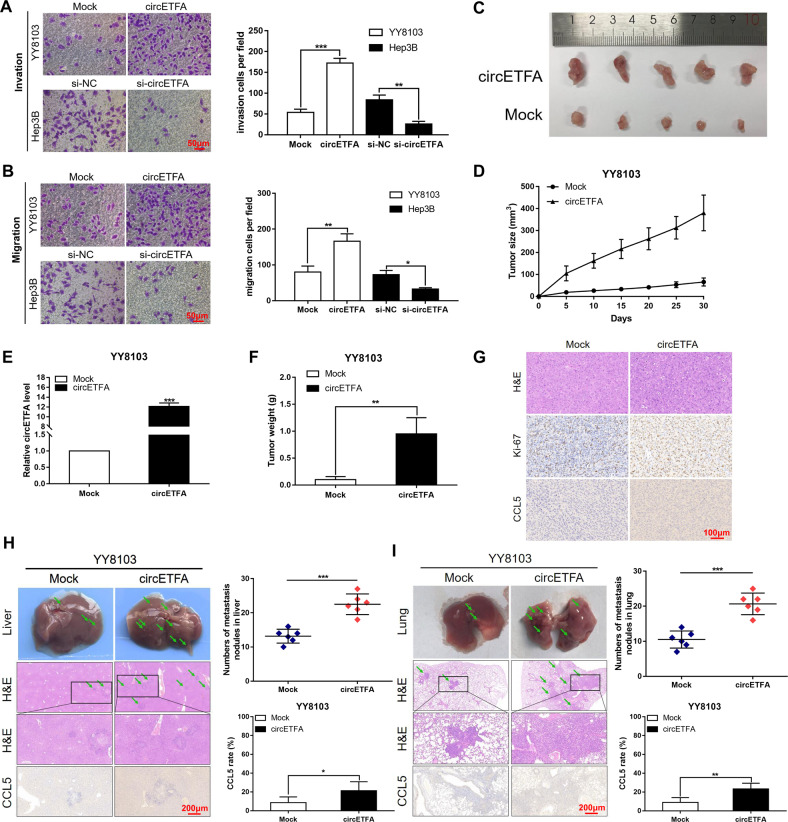
CircETFA promotes HCC progression in vivo. **A**, **B** Transwell assays were used to detect the influence of circETFA on the invasion and migration abilities of HCC cells. **C** The YY8103 cell line that stably overexpressed circETFA was subcutaneously implanted in nude mice. **D** Nude mice inoculated with YY8103/circETFA cells had faster tumor growth than those inoculated with YY8103/Mock cells. **E** CircETFA expression in tumors from YY8103/circETFA cells was higher than that from the Mock group after 30 days. **F** The weight of tumors from YY8103/circETFA cells was higher than that from the Mock group after 30 days. **G** IHC analysis of tumors of the YY8103/circETFA and YY8103/Mock groups. **H**, **I** Liver metastasis model and lung metastasis model were constructed to determine the influence of circETFA on HCC cell metastasis in vivo. The data are shown as mean ± SD. *P < *0.05*, **P < 0.01, ***P < 0.001.

Next, we used Transwell analysis to detect whether circETFA affects the invasion and migration of HCC. The results showed that the overexpression of circETFA significantly promoted the invasion and migration of HCC, while si-circETFA had the opposite effect (Fig. 4A, B). In summary, circETFA can promote the malignant phenotype of HCC in vitro.

### CircETFA promotes HCC cell growth and metastasis in vivo

To determine the biological role of circETFA in vivo, we subcutaneously implanted the YY8103 cell line stably overexpressing circETFA into nude mice (Fig. 4C). After 30 days, we found that the tumor growth of nude mice inoculated with YY8103/circETFA cells was faster than that of nude mice inoculated with YY8103/Mock cells (Fig. 4D). At the same time, circETFA expression and tumor weight were higher in tumors derived from YY8103/circETFA cells (Figs. 4E, F). Furthermore, immunohistochemical (IHC) analysis showed that Ki-67 expression in the YY8103/circETFA group was higher than that in the YY8103/Mock group (Figs. 4G and S1H). Subsequently, we also constructed a liver metastasis model by injecting YY8103 cells into the spleen and a lung metastasis model using tail vein injection in nude mice, with the purpose of studying the effect of circETFA on tumor metastasis. The results showed that mice injected with YY8103/circETFA cells had significantly more liver/lung metastatic nodules than the control group (Fig. 4H, I). In conclusion, circETFA overexpression contributes to tumor progression in vivo.

### CircETFA promotes tumor development by up-regulating CCL5 expression in HCC

**Fig. 5:**
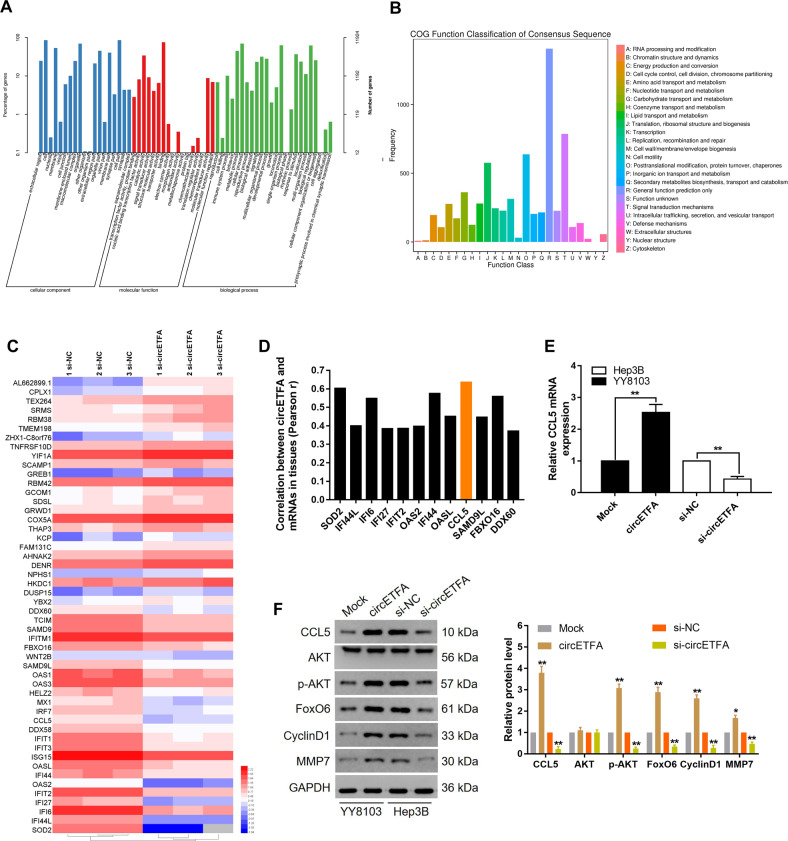
CircETFA upregulates CCL5 to affect PI3K/Akt signaling pathway. **A** GO analysis of the RNA-seq results. **B** Statistical results of the functional COG. **C** Heat maps were generated to show the FPKM values of the 50 most changed mRNAs converted by log_10_. **D** Relationship between circETFA expression and mRNAs levels in HCC tissues. **E** Detect changes in CCL5 expression by qRT-PCR after overexpression or knockdown of circETFA. **F** The protein levels of CCL5, Akt, p-Akt, FoxO6, CyclinD1, and MMP7 were measured by western blotting. The data are shown as mean ± SD. *P < *0.05*, **P < 0.01, ***P < 0.001.

To determine the mechanism by which circETFA promotes HCC, we used RNA transcriptome sequencing to evaluate the overall effect of circETFA knockdown. As shown in Supplementary Table S2, 186 genes in Hep3B cells were upregulated and 292 genes were downregulated (FC ≥ 1.5, *P* ≤ 0.05). Gene ontology (GO) analysis showed that the most obvious molecular functions involved catalytic activity and signal transducer activity, and the most obvious biological processes involved included cellular processes and biological regulation (Fig. 5A). The statistical results of the functional classification of orthologous groups (COG) indicated that the most obvious metabolic or physiological biases involved were signal transduction mechanisms and post-translational modifications (Fig. 5B). In terms of up and downregulation, the 50 most variable mRNA FPKM values were converted by log_10_ (Fig. 5C). We detected the expression of the most downregulated mRNAs in HCC tissues and analyzed the correlation between them and circETFA. The results showed that the Pearson r between the expression of CCL5 mRNA and circETFA was the highest (r = 0.6355) (*P* < 0.05) (Fig. 5D). C-C motif chemokine ligand 5 (CCL5) is a chemokine gene (NCBI, gene ID: 6352) on chromosome 17. CCL5 stimulates the expression of CCR5 in HCC, and this interaction promotes the malignant phenotype [30]. In this regard, we tested the expression level of CCR5 in HCC tissues and found it to be highly expressed (Fig. S2A). At the same time, we have also verified that CCL5 can promote the HCC process (Fig. S2B, C). In addition, CCL5 can activate PI3K/Akt and NF-κB pathways and FoxO6 is considered a key downstream molecule of PI3K/Akt signaling [31, 32]. Next, we explored whether circETFA regulates the expression and/or activity of key components of the PI3K/Akt signaling pathway by regulating CCL5. At the mRNA level, circETFA knockdown reduced the expression of CCL5 mRNA, while overexpression of circETFA increased the expression of CCL5 mRNA (Fig. 5E). At the protein level, after circETFA was overexpressed, CCL5 was upregulated and p-Akt, FoxO6, and the downstream effectors of FoxO6 (CyclinD1 and matrix metalloproteinase [MMP]−7) were also upregulated, while knocking down circETFA produced the opposite result (Fig. 5F) [33]. At the animal level, immunohistochemical analysis of subcutaneous tumors, liver and lung metastasis models in nude mice showed that CCL5 was increased in the YY8103/circETFA group (Figs. 4G–I and S1H). These findings indicate that CCL5 upregulation by circETFA in HCC helps increase the phosphorylation and activation of key components of the PI3K/Akt pathway.

**Fig. 6:**
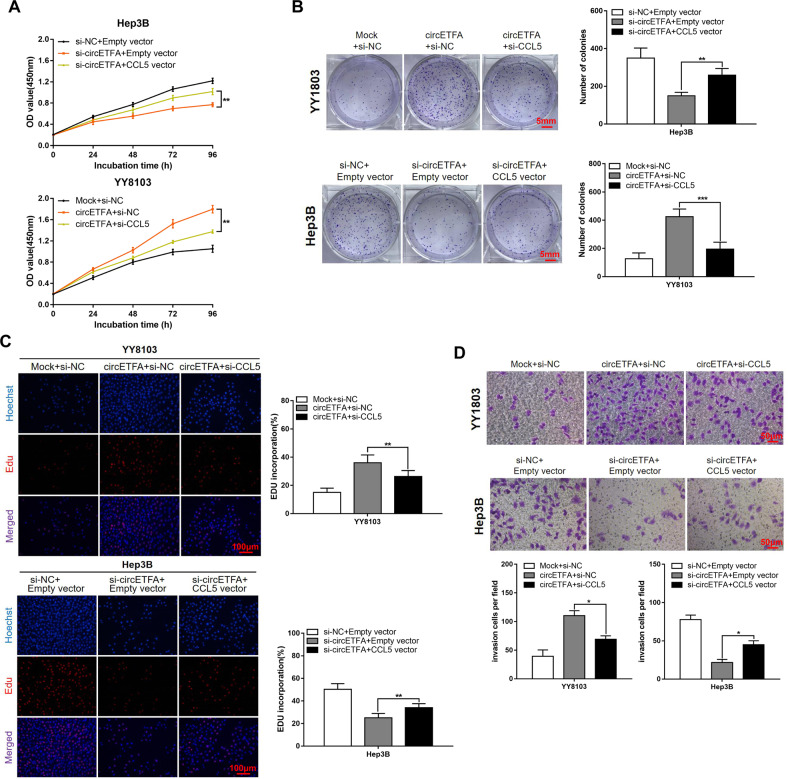
CircETFA promotes HCC by up-regulating CCL5 expression. **A**–**C** CCK-8 analyses, clone formation experiments, and EdU assays showed that co-transfection partially attenuated the circETFA-induced increase in proliferation. **D** Transwell assays showed that co-transfection partially inhibited the circETFA-induced increase in migration. The data are shown as mean ± SD. *P < *0.05*, **P < 0.01, ***P < 0.001.

To study whether circETFA upregulates CCL5 to promote the malignant HCC phenotype, YY8103 cells stably expressing circETFA were co-transfected with si-CCL5. CCK-8 (Fig. 6A), clone formation (Fig. 6B), EdU (Fig. 6C), and Transwell analyses (Fig. 6D) proved that co-transfection can partially attenuate the proliferation and invasion ability enhanced by circETFA. These results indicate that circETFA promotes the malignant phenotype of HCC by upregulating the expression of CCL5.

### CircETFA regulates CCL5 by competitively binding hsa-miR-612

**Fig. 7:**
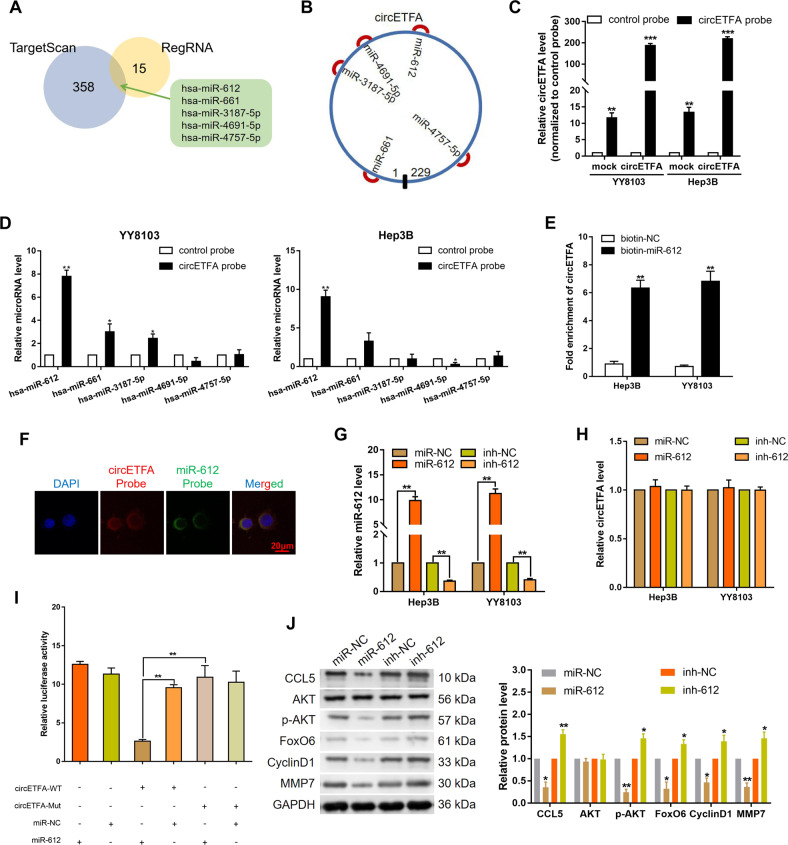
CircETFA regulates CCL5 by directly sponging hsa-miR-612. **A**, **B** The Bioinformatics prediction results showed that hsa-miR-612, hsa-miR-661, hsa-miR-3187-5p, hsa-miR-4691-5p, and hsa-miR-4757-5p contained relevant complementary sites to circETFA-miRNA-CCL5. **C** Lysates prepared from HCC cells overexpressing circETFA were subjected to RNA pull-down experiments, and the pull-down efficiency was confirmed by qRT-PCR. **D** The relative levels of 5 candidate miRNAs in HCC cell lysates were examined by qRT-PCR after RNA pull-down experiments using circETFA probe. **E** Enrichment level of circETFA was detected after pull-down assay using biotin-coupled miR-612 mimics. **F** Co-localization between hsa-miR-612 and circETFA was observed by FISH assay in Hep3B. **G** The efficiency of hsa-miR-612 mimic and inhibitor. **H** Hsa-mir-612 did not affect the expression of circETFA in HCC cell lines. **I** Hsa-miR-612 mimics and the circETFA reporter genes were co-transfected into 293 T cells for dual-luciferase reporter experiments. **J** Hsa-miR-612 inhibitor increased CCL5 protein levels, while hsa-miR-612 mimics had the opposite effect. The data are shown as mean ± SD. *P < *0.05*, ** *P* < 0.01, ***P < 0.001.

In the cytoplasm, circRNAs often act as sponges for miRNAs to enhance the translation of targeted genes because they have many miRNA binding sites [34–36]. In this study, we have confirmed that circETFA is mainly cytoplasmic (Fig. 2F, G), so we speculate that it may act as an miRNA sponge in HCC cells. To test this conjecture, we used the online software programs TargetScan Release 7.2 (http://www.targetscan.org/vert_72/) and RegRNA 2.0 (http://regrna2.mbc.nctu.edu.tw/index.html). The results showed that hsa-miR-612, hsa-miR-661, hsa-miR-3187-5p, hsa-miR-4691-5p, and hsa-miR-4757-5p contained relevant complementary sites for circETFA-miRNA-CCL5 (Supplementary Table S3) (Fig. 7A, B). To validate the predicted results, we performed a biotin-conjugated probe pull-down experiment, which showed that overexpression of circETFA significantly increased the pulldown efficiency (Fig. 7C). qRT-PCR analysis revealed that hsa-mir-612 was the most significantly miRNA pulled down by the circETFA probe in both YY8103 and Hep3B cells (Fig. 7D). At the same time, by a pull-down assay using biotin-coupled miR-612 mimics, we observed obvious enrichment of circETFA compared with the control (Fig. 7E). In addition, the double FISH assay indicated the co-localization of circETFA and hsa-mir-612 (Fig. 7F). In this regard, we further detected the expression of hsa-miR-612 in HCC tissues and analyzed the correlation between circETFA and hsa-miR-612. As shown in Figure S2D, in the HCC tissues, the expression level of circETFA was not correlated with the level of hsa-miR-612 (*P* = 0.1230). Subsequently, we tested the efficiency of hsa-miR-612 mimics and inhibitors (Fig. 7G). We found that hsa-miR-612 mimics or inhibitors did not change the expression of circETFA in HCC cell lines, and the change of circETFA also did not affect the expression of hsa-mir-612 (Figs. 7H and S2E). To further test our hypothesis, we conducted a dual luciferase reporter gene experiment. First, we constructed WT and circETFA mutant fragments of the 3’-UTR luciferase reporter gene (Fig. S2F). Subsequently, we co-transfected the hsa-miR-612 mimic with the circETFA reporter gene into 293 T cells and observed that the activity of the WT luciferase reporter gene was significantly reduced (Fig. 7I). Taken together, these data demonstrate that circETFA acts as a miRNA sponge for hsa-miR-612 in HCC.

**Fig. 8:**
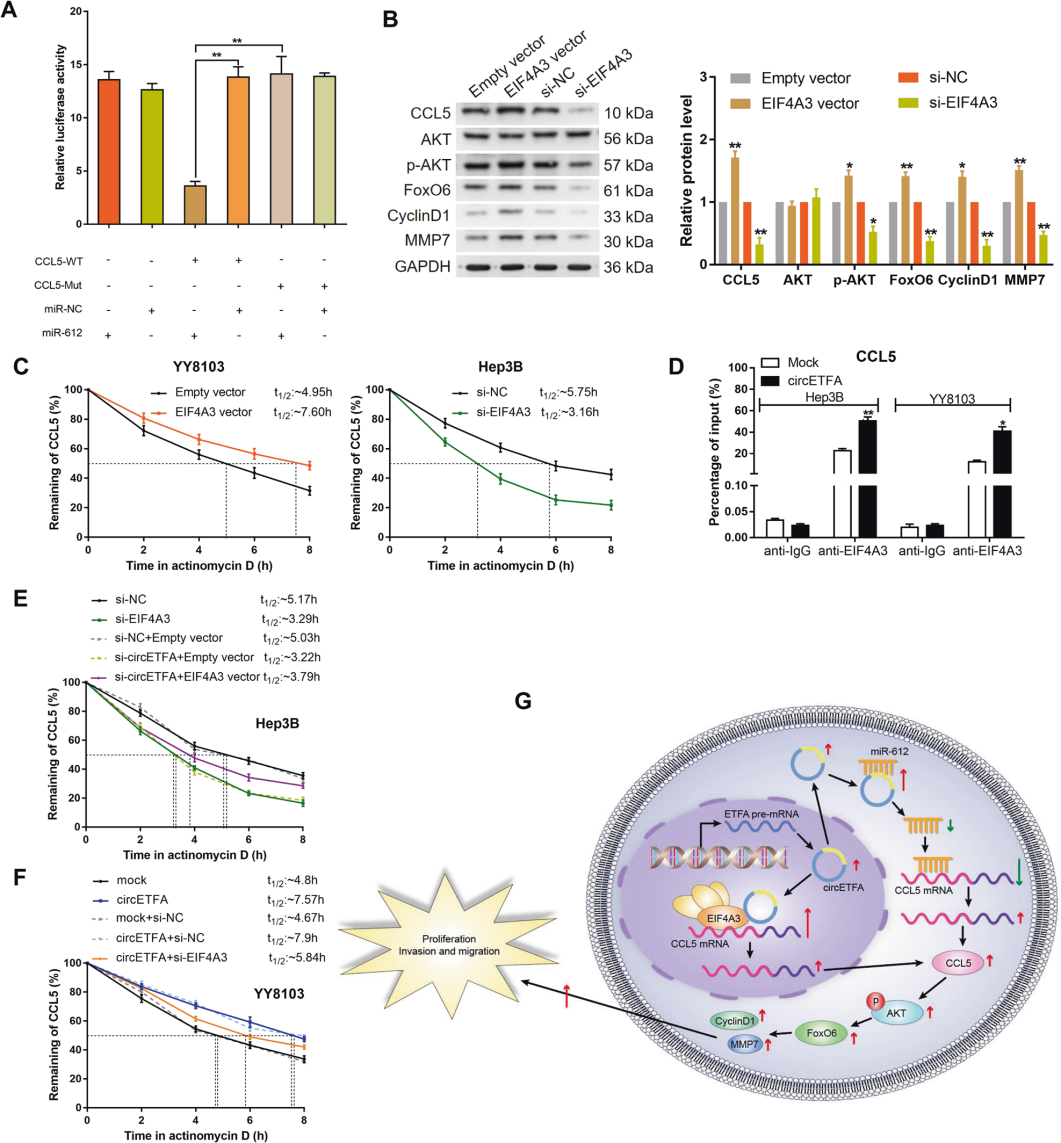
CircETFA regulates CCL5 mRNA by recruiting EIF4A3. **A** The activity of the luciferase reporter gene of wild-type or mutant CCL5 3’-UTR was tested in dual-luciferase reporter experiments. **B** The protein levels of CCL5, Akt, p-Akt, FoxO6, CyclinD1, and MMP7 were measured after transfecting HCC cells with EIF4A3 vector and si-EIF4A3. **C** The half-life of CCL5 was detected after transfecting HCC cells with EIF4A3 vector and si-EIF4A3. **D** RIP was conducted to determine whether circETFA can recruit EIF4A3 to CCL5 mRNA. **E**, **F** The half-life of CCL5 was detected after co-transfecting HCC cells with si-circETFA/EIF4A3 vector or circETFA/si-EIF4A3. **G** CircETFA increases the half-life of CCL5 by recruiting EIF4A3 and acting as a sponge of hsa-miR-612. CircETFA can upregulate CCL5 in HCC cells, thereby regulating key downstream effectors such as the PI3K/Akt pathway and FoxO6 to promote HCC cell proliferation, invasion, and migration. The data are shown as mean ± SD. *P < *0.05*, **P < 0.01, ***P < 0.001.

We wanted to further examine the relationship between hsa-miR-612 and CCL5. First, we detected the expression of hsa-miR-612 in HCC tissues and found that the expression level of CCL5 negatively correlated with the level of hsa-miR-612 (*P* = 0.0058, Pearson r = −0.4919) (Fig. S3A). Additionally, hsa-miR-612 had an inhibitory effect on CCL5, both at the mRNA and protein levels (Figs. S3B and 7J). Furthermore, the hsa-miR-612 inhibitor partly reversed the decrease in CCL5 induced by si-circETFA (Fig. S3C). We then mutated the CCL5 3’-UTR binding site (Fig. S2F) and observed reduced activity of the luciferase reporter gene for the WT CCL5 3’-UTR (Fig. 8A). These data indicate that circETFA can act as a sponge for hsa-miR-612 in HCC cells, thereby regulating CCL5 expression. We used the luciferase reporter gene detection method to further study the relationship between circETFA, hsa-miR-612, and CCL5. After overexpression of circETFA, the expression of the luciferase reporter gene increased, but the co-transfection of circETFA and hsa-miR-612 offset this effect of the WT CCL5 sequence, and mutation of the hsa-miR-612 binding site caused the effect to disappear (Fig. S3D). In conclusion, circETFA sponges hsa-miR-612 to upregulate the expression of CCL5 to promote the malignant phenotype of HCC (Fig. 8G).

### CircETFA prolongs the half-life of CCL5 mRNA by recruiting EIF4A3

As shown in Fig. 2F, G, circETFA is also partially distributed in the nucleus. A large number of studies have shown that certain circRNA can “absorb” certain things, such as RBPs [37]. Therefore, we conducted a bioinformatics analysis in the Circular RNA Interactome (https://circinteractome.nia.nih.gov/index.html) to predict the RBPs that may interact with circETFA. The results showed that circETFA and EIF4A3 had the most, that is, five putative binding sites (Supplementary Table S4). As the core component of the exon junction complex (EJC), EIF4A3 can regulate and monitor events such as mRNA subcellular localization and half-life [38, 39]. EIF4A3 mainly contacts the ribose phosphate backbone of mRNA [38, 39]. First, we performed RIP and qRT-PCR, which proved that EIF4A3 binds to circETFA (Fig. S3E). In addition, the overexpression of EIF4A3 led to the upregulation of circETFA, while the knockdown of EIF4A3 led to the downregulation of circETFA (Fig. S3F). At the mRNA level, we observed that si-EIF4A3 reduced the expression of CCL5 mRNA, while the overexpression of EIF4A3 resulted in the upregulation of CCL5 mRNA (Fig. S3G). At the protein level, overexpression of EIF4A3 resulted in an increase in CCL5, while the knockdown of EIF4A3 decreased CCL5 expression (Fig. 8B). Subsequently, we demonstrated that overexpression of EIF4A3 can partially reverse the decline of CCL5 induced by si-circETFA (Fig. S3C). As shown in Fig. S3H, the expression level of EIF4A3 was positively correlated with the expression of CCL5 (*P* = 0.0114, Pearson r = 0.4556). We also found that the half-life of CCL5 mRNA was positively correlated with EIF4A3 expression (Fig. 8C). Therefore, we hypothesized that circETFA can increase the half-life of CCL5 mRNA by recruiting EIF4A3. To test this hypothesis, we conducted RIP experiments by overexpressing circETFA and observed an increase in the binding of EIF4A3 to CCL5 mRNA (Fig. 8D). Then, Hep3B cells knocked down circETFA were co-transfected with EIF4A3 vector and the half-life of CCL5 mRNA reduced by si-circETFA was further reduced to a certain extent (Fig. 8E). Si-EIF4A3 partially attenuated the prolongation of the half-life of CCL5 induced by circETFA (Fig. 8F). Therefore, in the nucleus, circETFA can regulate CCL5 through EIF4A3, thereby promoting the malignant HCC phenotype (Fig. 8G).

## Discussion

In the present study, we discovered and identified circETFA and observed that circETFA is abnormally high in HCC and is associated with a poor prognosis. Moreover, in vivo and in vitro functional tests have shown that highly expressed circETFA can promote the malignant phenotype of HCC. We performed RNA transcriptome sequencing and proved that circETFA can promote the process of HCC by upregulating CCL5 and regulating PI3K/Akt signaling. We predicted through bioinformatics that circETFA and CCL5 could compete to bind with hsa-miR-612. Subsequently, a series of experiments, including RNA pull-down, dual luciferase reporter gene, correlation analysis, and other experiments, further proved that circETFA can act as a sponge for hsa-miR-612 to prevent the inhibitory effect of hsa-miR-612 on CCL5. Additionally, through database prediction, we found that EIF4A3 has the most binding sites that may bind to circETFA. Subsequent studies have shown that EIF4A3 can act as an RBP to bind with circETFA and extend the half-life of CCL5 mRNA.

The identification and study of a large number of circRNAs as a novel type of non-coding RNA, which are considered to be associated with a variety of cancers [40]. For example, circ-CCAC1 plays a crucial role in the occurrence and development of cholangiocarcinoma [41]. The circSDHC / miR-127-3p / CDKN3 / E2F1 axis promotes renal cell carcinoma progression [18]. In the present study, circETFA proved to be a promising biomarker for HCC. Increasing attention has also been paid to the specific mechanisms by which circRNAs exert their effects. Initially, researchers generally believed that circRNAs exert their effects by acting as sponges for miRNAs [42]. Many studies have also proved this notion, for example, circ_001621 promotes osteosarcoma progression by sponging miR-578 [43]. Our results also suggest that circETFA, which is mainly distributed in the cytoplasm, can also promote HCC progression by competitively binding to miR-612. However, more research findings have shown that some circRNAs can also bind to RBPs to play regulatory roles [44]. In melanoma, CDR1as does not act by sponging miR-7 classically but by binding to IGF2BP3 [45]. CircYAP can inhibit myocardial fibrosis by binding to TMP4 and ACTG [46]. Interestingly, through a combination of biochemical prediction and experimental validation we found that circETFA interacts with EIF4A3. EIF4A3 is a core protein of EJC, and EJC is an important component of messenger ribonucleoprotein particles (mRNPs). The remodeling of mRNPs can affect the processing of precursor mRNAs in advanced organisms [47]. Therefore, EJC affects gene expression steps, such as the splicing of eukaryotic mRNA precursors and the nuclear transport and degradation of mRNA [48, 49]. In addition, there are reports that EIF4A3 can promote circRNA biogenesis. For example, EIF4A3 can bind to MMP9 mRNA transcripts and promote the formation of circMMP9 [50]. In future studies, we will demonstrate whether EIF4A3 can promote circETFA formation and specific regulatory mechanisms. In addition, circRNAs were previously thought not to be translationally competent; however, current studies have also demonstrated the ability of partial circRNAs to be translated into polypeptides [51]. For example, Circ-AKT3 can encode AKT3-174aa, which plays a negative regulatory role in regulating PI3K / AKT signal intensity [52]. In this study, we predicted using database and found that circETFA is not translationally competent, but further experiments are needed to prove this conclusion.

CCL5, also known as RANTES, is a chemokine associated with tumor-infiltrating lymphocytes and is secreted by T lymphocytes, macrophages, and certain tumor cells. CCL5 stimulates CCR5 expression in HCC, and upon binding to CCL5, the CCR5 heterotrimeric G protein dissociates into two subunits:α subunit and βγ subunit [30, 53, 54]. The alpha subunit inhibits the adenylyl cyclase. The βγ subunit can activate PLC and PI3K and can cause enzyme activation by phosphorylating the ser473 residue of Akt through a PI3K dependent signaling pathway [55, 56]. Halama et al. [57] demonstrated that the CCL5/CCR5 axis promotes the malignant phenotype of HCC cells. Our results also confirmed that CCL5 and CCR5 were highly expressed in HCC tissues, and circETFA promoted tumor development by upregulating the expression of CCL5. In addition, CCL5 expressed by cancer cells plays an important role in remodeling the tumor microenvironment [58–60], whether circETFA can participate in regulating tumor immune evasion by regulating CCL5 is also worthy of investigation.

## Conclusion

In summary, we have identified and proved circETFA can upregulate CCL5 and affect the PI3K/Akt signaling pathway to promote HCC progression. The specific molecular mechanism is that circETFA can not only sponge hsa-miR-612 to save CCL5 from being silenced and degraded but also recruit EIF4A3 to extend the half-life of CCL5, which ultimately leads to the upregulation of CCL5. Based on our data, we suggest that circETFA may be a new biomarker and therapeutic target for patients with HCC.

## Materials and methods

### Patient samples

56 pairs of HCC cancer tissues and adjacent normal tissues were sampled from HCC patients at the Nanjing First Hospital, confirmed by professional pathologists, and kept in liquid nitrogen. The inclusion criteria were: patients with complete survival data and the enrolled patients without prior chemotherapy, radiation therapy, immunotherapy, and so on. According to the principles of the Declaration of Helsinki, all patients provided written informed consent, and the protocol was approved by the Ethics Committee of Nanjing Medical University (Nanjing, Jiangsu).

### Quantitative reverse transcription-polymerase chain reaction (qRT-PCR) and RNase R resistance analysis

Total RNA was extracted using the TRIzol reagent (Takara, Japan), and 2 mg of total RNA was incubated with or without 3 U/mg RNase R (Epicentre Technologies, Madison, WI, USA) for 30 min at 37 °C. Next, we reverse-transcribed RNA to obtain cDNA. Then, they were analyzed by qRT-PCR using specific primers. An ABI 7500 real-time PCR system (Applied Biosystems, Foster City, CA, USA) and SYBR Green Master Mix (Takara, Shiga, Japan) were used to measure the relative RNA expression levels. All the primer sequences are listed in Supplementary Table S5.

### Fluorescence in situ hybridization (FISH) assay

Specific probes were designed for the circETFA sequence (Supplementary Table S5). After collecting the cells, they were fixed in formaldehyde and then washed with phosphate-buffered saline (PBS), and dehydrated with ethanol. The hybridization buffer was mixed with FISH probe and cells and then incubated with a tyramide-conjugated Alexa 488 fluorochrome TSA kit.

### Isolation of cytoplasmic and nuclear RNA

The nuclei and cytoplasm of HCC cells were isolated using a PARIS kit (Life Technologies, Carlsbad, CA, USA). Reverse transcription of the extracted RNA was performed to obtain the cDNA for qRT-PCR. The primer sequences used are listed in Supplementary Table S5.

### Cell culture

Human normal liver HL7702 and HCC cell lines (Cell bank of the Chinese Academy of Sciences, Shanghai, China) were cultured in Dulbecco’s modified Eagle’s medium (Gibco, Grand Island, NY, USA), supplemented with 10% fetal bovine serum (FBS, Gibco) and 1% penicillin and streptomycin. The cells were maintained in a cell incubator containing 5% CO2 at 37 °C.

### Construction of a cell line stably expressing circETFA

The Hanheng Company (Shanghai, China) constructed a lentiviral vector to stably overexpress circETFA. YY8103 cells were cultured, transfected, and screened with puromycin.

### Plasmid construction and cell transfection

We transfected small interfering RNAs (siRNAs) and plasmids into HCC cells using Lipofectamine 3000 (Invitrogen, Carlsbad, CA, USA) and p3000. The siRNA sequences designed for circETFA, C-C motif chemokine ligand 5 (CCL5), eukaryotic initiation factor 4A3 (EIF4A3), and mimics and inhibitors of hsa-miR-612 are listed in Supplementary Table S5. The cDNAs of CCL5 and EIF4A3 were cloned into the pcDNA3.1 vector.

### Cell viability assay

Cell Counting Kit-8 (CCK-8) assay performed according to the manufacturer’s instructions (Dojindo, Kumamoto, Japan) was used to measure cell proliferation. Absorbance at 450 nm was measured using a microplate reader (BioTek, Winooski, VT, USA).

### Colony formation assay

Cells were distributed evenly in each well of the six-well plates and the plates were incubated for 2 weeks. The plate was then removed from the incubator, fixed with methanol, stained with crystal violet, and photographed.

### 5-Ethynyl-2’-deoxyuridine (EdU) assay

The cells used for the experiments were pre-seeded in 24-well plates. The EdU solution was prepared, added to a 24-well plate, and incubated for 2 h. The cells were then fixed with formaldehyde and stained with EdU for 30 min. Reagent F was used for nuclear staining, and fluorescence was observed under a microscope (Olympus, Tokyo, Japan) to record the positive rate of EdU staining.

### Cell invasion and migration assays

According to the study design, 10,000 HCC cells were evenly dispersed in the upper Transwell chamber (Corning, Corning, NY, USA), and serum-free medium was added. A complete medium containing 10% FBS was added to the lower chamber. After 24 h, fixed with methanol, and stained with crystal violet. The results were observed using an inverted microscope.

### Animal models

Based on the experimental design, we set circETFA and control groups, stably overexpressed by HCC cells in 4-week-old nude mice (Animal Center of Nanjing University, Nanjing, China), to perform cell proliferation assays. Each nude mouse was subcutaneously inoculated with 3 × 10^6^ cells and observed every 5 days to record tumor size and volume. The asphyxiated nude mice were sacrificed and analyzed after 30 days. For the liver metastasis model, 2 × 10^6^ cells were injected into the distal spleen of each nude mouse. After eight weeks, the mice were sacrificed and dissected. For the lung metastasis model, 5 × 10^5^ cells were injected into the tail vein of each nude mouse. After six weeks, the mice were sacrificed and dissected. This study was conducted in strict accordance with the guidelines of the National Institutes of Health (NIH) and the Animal Experimental Ethics Committee of Nanjing Medical University.

### Immunohistochemical analysis

For immunohistochemical studies, samples were incubated overnight at 4 °C with anti-Ki67 and anti-CCL5 antibodies. After incubated with secondary antibodies, the sections were stained with the secondary, washed, dehydrated, treated with a transparent medium, and fixed on a glass slide before observation under a microscope.

### Western blot

Cells were processed to prepare protein samples, and then loading buffer was added in proportion before denaturing by boiling. The protein samples were separated by gel electrophoresis and then transferred to polyvinylidene fluoride membranes. At room temperature, the membrane was blocked with 5% skimmed milk powder on a shaker for 1 h and then incubated with primary antibodies (Abcam, USA) overnight at 4 °C. After incubation with milk, primary antibody and secondary antibody, the films were treated with enhanced chemiluminescence reagents. Band intensity was measured by densitometry.

### RNA pulldown

Biotin probes were designed for circETFA and miR-612. The probe sequences are listed in Supplementary Table S5. The probe was incubated with C-1 magnetic beads (Life Technologies) at 25 °C for 2 h. The cell lysate with circETFA probe or oligonucleotide probe was incubated at 4 °C overnight. After eluting the RNA mixture bound to magnetic beads, qRT-PCR was performed using RNeasy Mini Kits (QIAGEN, Hilden, Germany).

### Dual-luciferase reporter gene assay

Wild-type (WT) and mutant fragments corresponding to the 3’-untranslated region (UTR) in circETFA or CCL5 that may bind to hsa-miR-612 were designed and inserted into a reporter vector (Hanheng, Shanghai, China), and hsa-miR-612 mimic or inhibitor and circETFA/CCL5 WT or mutant reporter genes were then co-transfected into 293 T cells. Luciferase activity in the co-transfected cells was detected using a luciferase reporter assay system (Promega, Madison, WI, USA).

### RNA immunoprecipitation (RIP)

We purchased RBP immunoprecipitation kits (17–700, Merck, Millipore, Billerica, MA, USA) to study the relationship between circETFA and EIF4A3. We prepared HCC cell lysates for incubation with RIP buffer. The EIF4A3-associated RNA mixture was analyzed by qRT-PCR.

### Statistical analysis

Significant differences were estimated by Student’s *t*-test, Chi-square test, or one-way analysis of variance, as appropriate. Pearson correlation coefficients were calculated to assess correlations between variables. Differences were considered significant at *P* < 0.05.

## Supplementary information

SUPPLEMENTAL MATERIAL

Supplementary Table S1

Supplementary Table S2

Supplementary Table S3

## Data Availability

The data sets used in the current study are available in the GEO database (ID: GSE166678).
