# Supplementary material for: CircETFA upregulates CCL5 by sponging miR-612 and recruiting EIF4A3 to promote hepatocellular carcinoma
Source: Cell Death Discov. 2021 Oct 29;7:321. doi: 10.1038/s41420-021-00710-x (PMC8556257; doi:10.1038/s41420-021-00710-x)
Supplement: Supplementary file 1 — SUPPLEMENTAL MATERIAL [file 41420_2021_710_MOESM1_ESM.pdf]

Supplemental Material

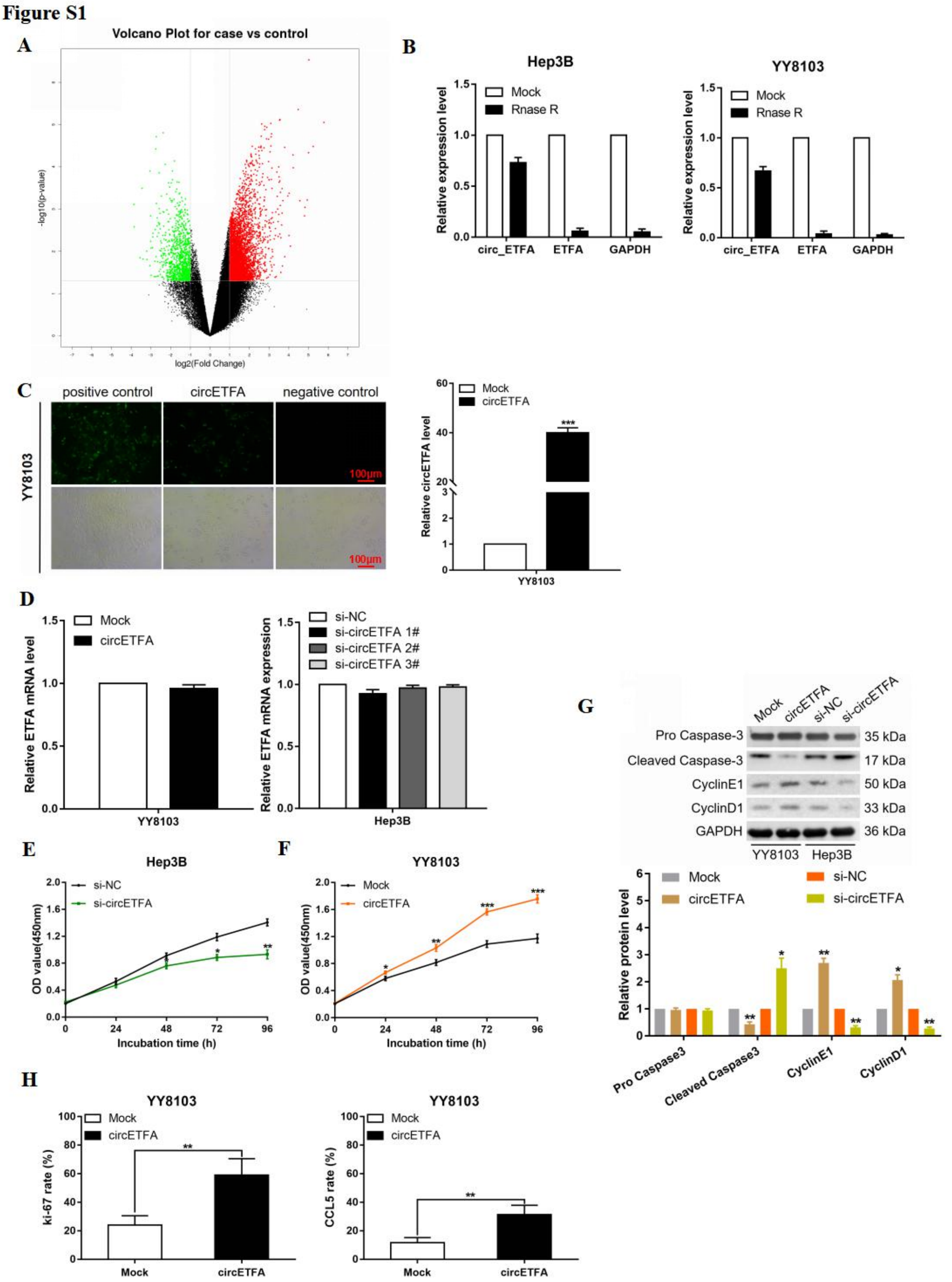

Figure S1. Identification and characterization of circETFA.

(A) The volcano plot showed that the circRNAs were significantly upregulated and downregulated. (B) RNase R tolerance tests excluded trans-splicing and genome rearrangements. (C) The lentivirus infection system was used to establish a YY8103

cell line stably overexpressing circETFA. (D) Overexpression or knockdown of circETFA had no effect on linear mRNA expression. (E-F) CCK-8 detection on cell proliferation after overexpression or knockdown of circETFA. (G) Western blot analysis of apoptosis- and cell-cycle-related proteins in YY8103 and Hep3B that knocking down or overexpressing circETFA. (H) The positivity rates of Ki-67 and CCL5 in the immunohistochemistry of subcutaneous tumor formation. Data are shown as the mean ± SD. *$P < 0.05$, ** $P < 0.01$, *** $P < 0.001$

CircRNA, circular RNA; CCL5, C-C motif chemokine ligand 5; CCK-8, Cell Counting Kit-8

Figure S2

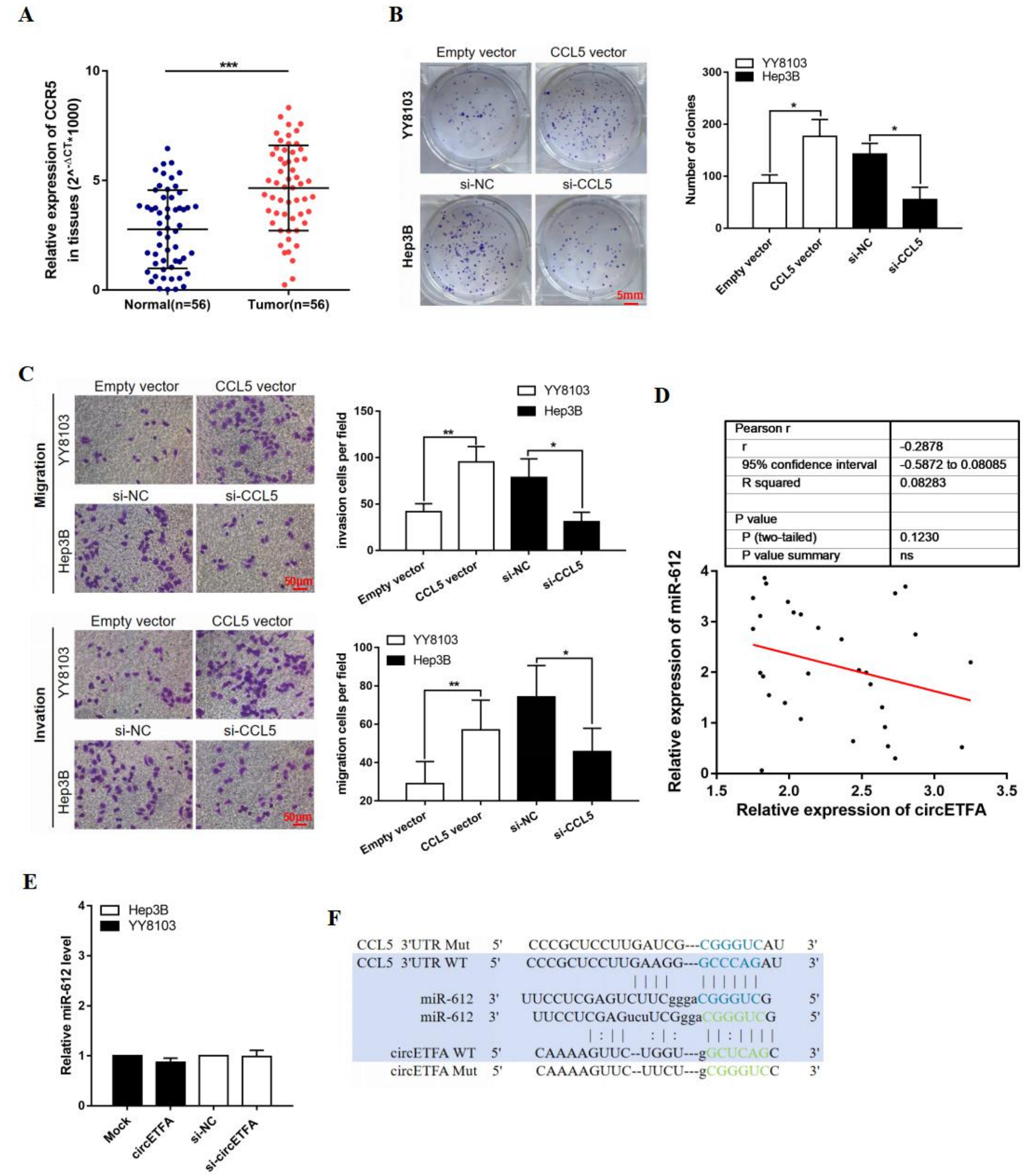

Figure S2. Effect of CCL5 on proliferation and invasion in vitro.

(A) CCR5 expression in HCC tissues. (B) Clone formation experiments showed that CCL5 promoted proliferation. (C) Transwell experiments showed that CCL5 promoted invasion and migration. (D) Correlation between circETFA and miR-612 in HCC tissues. (E) The expression of hsa-mir-612 was detected in HCC cells overexpressing or knocking down circETFA. (F) Wild-type and mutant

circETFA/CCL5 fragments of the 3'-UTR luciferase reporter gene were constructed. Data are shown as the mean ± SD. *$P < 0.05$, ** $P < 0.01$, *** $P < 0.001$

CCL5, C-C motif chemokine ligand 5; HCC, hepatocellular carcinoma; UTR, untranslated region

Figure S3

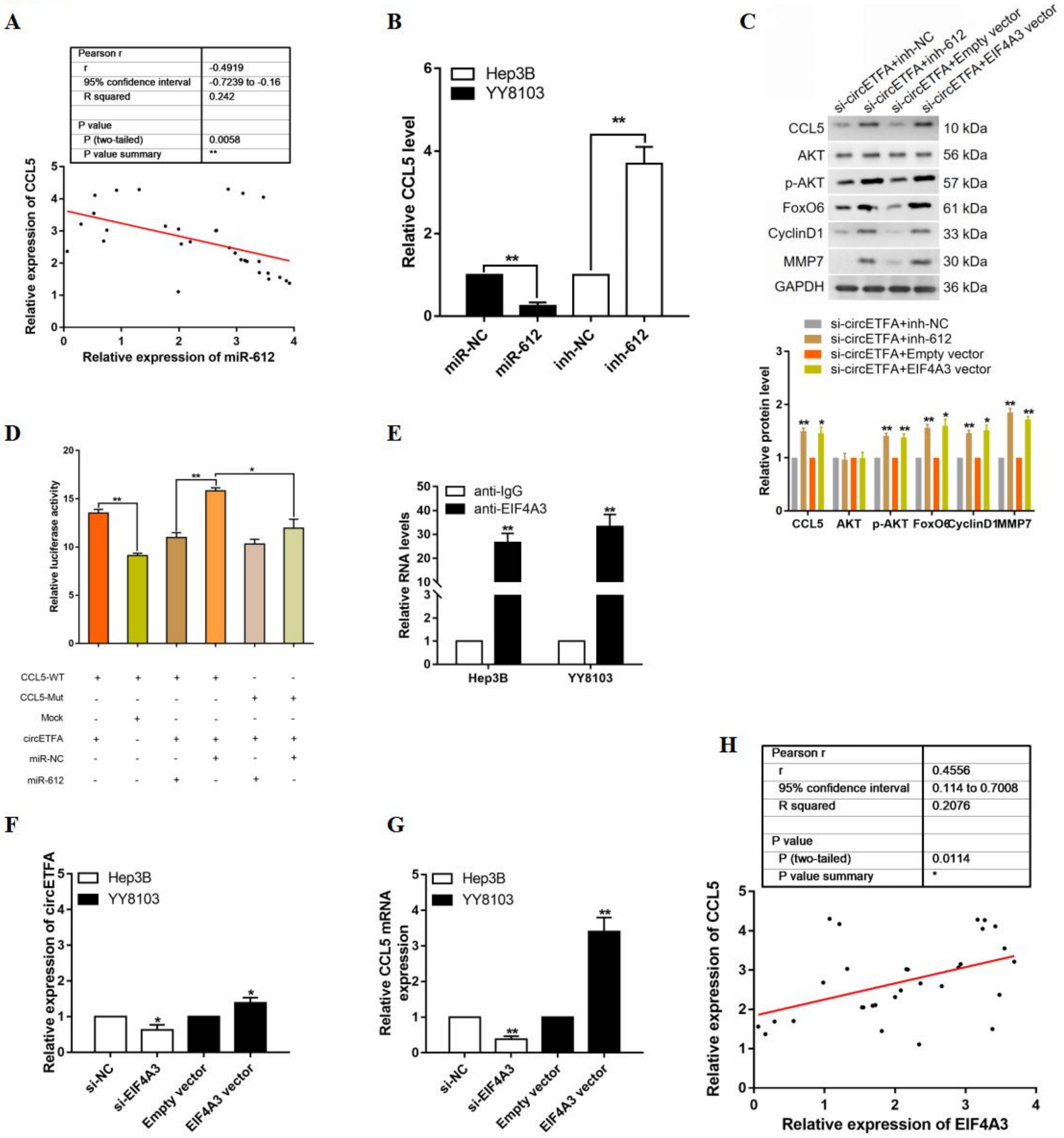

Figure S3. Correlation between circETFA and key molecules in HCC.

(A) The correlation between miR-612 and CCL5 in HCC tissues. (B) CCL5 expression in HCC cell treated with miR-612 mimics or inhibitors. (C)

Co-transfection partially rescued the expression of CCL5. (D) Luciferase reporter gene experiment among circETFA, miR-612, and CCL5. (E) RIP experiments for EIF4A3 were performed and co-predicted RNA was detected by qRT-PCR. The fold enrichment of circETFA in the Hep3B and YY8103 cells was matched using IgG as a control. (F-G) The effect of EIF4A3 on the expression of circETFA and CCL5. (H) Correlation between EIF4A3 and CCL5 in HCC organization. The data are shown as mean ± SD. $*P < 0.05$, ** $P < 0.01$, *** $P < 0.001$

HCC, hepatocellular carcinoma; CCL5, C-C motif chemokine ligand 5; EIF4A3, eukaryotic initiation factor 4A3; qRT-PCR, quantitative reverse transcription-polymerase chain reaction

Supplementary Table S1 (Excel)
Supplementary Table S2 (Excel)
Supplementary Table S3 (Excel)
Supplementary Table S4

CircRNA ID	hsa_circ_0036412
Genomic Length	3119 bp
Best Transcript	NM_000126 Primers
Samples	Ag04450, Bj, Gm12878, H1hesc, Helas3, Hepg2, Huvec, K562, Mcf7, Nhek
GenomicSeq	hsa_circ_0036412
RNA-binding protein sites matching to circRNAs	
RNA-binding protein sites matching flanking regions of circRNA	
RNA-binding Protein	# Tags
AGO2	2
C22ORF28	1
DGCR8	1
EIF4A3	5
FMRP	2
FUS	1
FXR2	1

HuR	3
IGF2BP1	1
IGF2BP2	1
IGF2BP3	1
PTB	1
TDP43	1
ZC3H7B	2

Supplementary Table S5

**Primers**	
hsa_circ_0036412 F	ACTTCCAGGCCTCATTGCTAC
hsa_circ_0036412 R	CGTGTGGCTGCAGTAATGGT
hsa_circ_0036411 F	AGCATGATGTGTACAAAGGCCTAC
hsa_circ_0036411 R	CTTTTGCTATGCCTGCTACTTTACA
hsa_circ_0036409 F	ACACATCTGTGCTGGAGCATCT
hsa_circ_0036409 R	TGCTTCTGAGTTGCCAAAATCAA
hsa_circ_0003620 F	TCGGAAAGGCCTCATTGCTAC
hsa_circ_0003620 R	TAAGCAGGACACTTCACCTCCA
hsa_circ_0028071 F	TTCTATGACCCTGACACCAGCA
hsa_circ_0028071 R	GGCTTCCCCTGTTCCCACATT
hsa_circ_0000437 F	AGATTGCCAGAAAAATATGCAGGA
hsa_circ_0000437 R	AAAGGCAGCAACACCCCATT
hsa_circ_0097182 F	ATGCCCAAGAGGGGACTTGA
hsa_circ_0097182 R	CTGCCATACCATGACCGTGC
hsa_circ_0028067 F	GGTTACATGCCCAAGAGGGG
hsa_circ_0028067 R	AGCTGACTACCAGGGCCATA
hsa_circ_0006852 F	GGGATGGGTTACATGCCCAA
hsa_circ_0006852 R	GCCACGATGCCGACTCTCTT
hsa_circ_0008092 F	TCAACACATTCAGCAGCAAGG
hsa_circ_0008092 R	TCCTTCTCCTGGCAATCTCACA
ETFA F	AGCTGAGCATGCAAATGATTCC
ETFA R	GCCACCTTGTCACATTTGGT
GAPDH F	GGGAGCCAAAAGGGTCAT
GAPDH R	GAGTCCTTCCACGATACCAA
CCL5 F	CTGCTTTGCCTACATTGCCC
CCL5 R	TCGGGTGACAAAGACGACTG
EIF4A3 F	AGGATATGGGGATTCTGCTCTC
EIF4A3 R	GTGAAATGACCCAAGACTACACA
U6 F	CTCGCTTCGGCAGCACA
U6 R	AACGCTTCACGAATTTGCGT
**siRNA sequence**	

si-circETFA 1#	GGCCTACTTCCAGGCCTCA
si-circETFA 2#	CCTACTTCCAGGCCTCATT
si-circETFA 3#	TCCCTGCCCTTTGACAGTG
si-EIF4A3	GGTCTGTCACTCATGGGTT
si-CCL5	GCAGAGGATCAAGACAGCA
**FISH probe**	
circETFA probe-1	ATGAGGCC+TGGAAG+TAGGCCT
circETFA probe-2	AATGAGGCC+TGGAAG+TAGGCC
circETFA probe-3	TAGCAA+TGAGGCC+TGGAAGTA
**Pulldown probe**	
circETFA probe-1	ATGAGGCC+TGGAAG+TAGGCCT
circETFA probe-2	AATGAGGCC+TGGAAG+TAGGCC
circETFA probe-3	TAGCAA+TGAGGCC+TGGAAGTA
hsa-miR-612	AAGGAGC+TCAGAAGCCC+TGCCCAGC
**Mimics and inhibitor**	
hsa-miR-612 mimics F	GCUGGGCAGGGCUUCUGAGCUCCUU
hsa-miR-612 mimics R	AAGGAGCUCAGAAGCCCUGCCCAGC
hsa-miR-612 inhibitor R	AAGGAGCUCAGAAGCCCUGCCCAGC
